# Genotoxicity of Anesthetics Evaluated *In Vivo* (Animals)

**DOI:** 10.1155/2015/280802

**Published:** 2015-06-24

**Authors:** Mariana G. Braz, Bensu Karahalil

**Affiliations:** ^1^Faculdade de Medicina de Botucatu, UNESP-Univ Estadual Paulista, Departamento de Anestesiologia, Distrito de Rubiao Junior, s/n, 18618-970 Botucatu, SP, Brazil; ^2^Toxicology Department, Faculty of Pharmacy, Gazi University, 06330 Ankara, Turkey

## Abstract

The anesthesia has been improved all over the years. However, it can have impact on health, in both patients and animals anesthetized, as well as professionals exposed to inhaled anesthetics. There is continuing effort to understand the possible effects of anesthetics at molecular levels. Knowing the effects of anesthetic agents on genetic material could be a valuable basic support to better understand the possible mechanisms of these agents. Thus, the purpose of this review is to provide an overview on the genotoxic potential, evaluated in animal models, of many anesthetics that have already been used and those currently used in anesthesia.

## 1. Introduction

Nowadays, technologies provide evaluation of possible toxic effects of anesthetics at cellular/molecular level, pointing out a new look in this field of study. Thus, genetic toxicology is gaining a lot of attention regarding the genotoxic evaluation of drugs, such as anesthetics.

The goal of this review is to update information about the possible genotoxic effects of the most used drugs in anesthesia all over the world, evaluated* in vivo*, that is, in animal models. Most of the genotoxic evaluations of anesthetics were performed in mammals (especially in rodents, but also in dogs and equines) and further in fruit fly and fish. The majority of the studies evaluated old-fashioned and also currently used volatile anesthetics, including the halogenated, but only a few have evaluated intravenous or local anesthetics.

The* in vivo* assays are especially relevant to assess genotoxic/mutagenic hazard in that the assay's responses are dependent upon* in vivo* Absorption, Distribution, Metabolism, and Excretion (ADME) and also on DNA repair processes. Thus, the animal model is ideal for simulating human's response during anesthesia, but without the surgical stress. Using animal model, it is easier to observe the possible genotoxic effect of anesthetics themselves instead of anesthesia in combination with surgical trauma on stress response, as occurs in clinical practice. It is known that surgery can lead to inflammation and oxidative stress, damaging genetic material [1].

The literature points out many reports that evaluated different types of anesthetics (used alone/single or in combination), with different doses, time of exposure, animal species, and endpoints evaluated. Therefore, the goal of this paper is to provide a review on the genotoxic potential of some new and old-fashioned anesthetics used in veterinary and human anesthesia practice. Herein, genotoxicity data of these agents are of special importance, particularly because of conflicting results so far.

Cytogenetic biomarkers are most frequently used and well-established endpoints in studies with their sensitivity for measuring exposure to genotoxic agents. Biomarkers of effect are indicators of a change in biologic function in response to a chemical exposure [2]. Thus, biomarkers of effect are more directly related to adverse effects/clinical response compared with biomarkers of exposure [3]. Biomarkers of early biologic effect include cytogenetic assays, such as sister chromatid exchanges (SCE), chromosomal aberrations (CA), and micronucleus (MN); the single cell-gel electrophoresis (SCGE), known as comet assay, is also a frequently used endpoint to evaluate DNA damage. The genotoxicity assays are of special concern since genotoxicity has gained widespread acceptance as an important and useful indicator of carcinogenicity [4, 5].

The conventional alkaline comet assay detects damage including double- and single-strand DNA breaks, alkaline-labile sites, DNA-protein, and DNA-DNA cross-linking that occurs rapidly on exposure. Furthermore, oxidative damage sites (using endonuclease III, endo III, and formamidopyrimidine glycosylase, Fpg) can also be detected. When exposure is discontinued, the primary lesions detected by this assay are most often repaired in a few minutes to hours without persistent genetic alterations. The comet assay belongs to the group of indicator tests (as opposed to mutagenicity tests) because it detects DNA damage that may result in mutations. Thus, this test also provides an index of the kinetics of DNA strand break repair and break excision repair. As an “early warning system,” the comet assay can detect very low levels of DNA damage. Thus, it provides few negative results on exposure to well-known genotoxins and fewer false positive results than other assays [6]. Since comet assay is a very sensitive tool, especially to detect DNA damage after chemical exposure that contributes to genetic instability and cancer in experimental researches, it has been used as a screening tool after* in vivo* exposure [7].

In relation to cytogenetic assays, SCE is a sensitive index of chromosome damage; it is the manifestation of interchanges between DNA replication products due to a consequence of DNA replication errors [8]. Despite the fact that molecular mechanisms underlying SCE formation are not well understood, they are thought to reflect DNA damage and/or DNA repair [9]. The CA analysis is a traditional method and is important for monitoring populations exposed to genotoxic agents. It can evaluate the whole genome to identify mutagenic and carcinogenic chemicals [10], and it has been found to be predictive of cancer. Another indicator for monitoring genetic damage is the MN assay. It can be applied to many biological materials such as peripheral blood cells, bone marrow, and other organs from animals exposed. The frequency of MN is reliable to chromosome loss and breakage. Thus, MN assay allows the detection of both clastogenic and aneugenic agents. An increased MN frequency predicts the risk of cancer in human population [11].

Genotoxicity tests including bacterial mutagenicity and* in vitro* and* in vivo* chromosome stability assays are mandatory by regulatory agencies worldwide prior to marketing, and it is mandatory for all the new drugs to be tested for their genotoxicity potential together with general toxicity testing [12]. In addition, regulatory agencies and scientific committees have been recommending the use of the* in vivo* comet assay to give support to data found in experimental or* in vitro* genotoxicity tests [13, 14].

As a result, the purpose of the present paper is to provide a comprehensive review on the genotoxic potential of a variety of anesthetics, based on our research.

## 2. Old-Fashioned Inhalation Anesthetics

Chloroform started to be used one year later than ether anesthesia (October 16, 1846, at the Ether Dome in Boston at Massachusetts General Hospital, USA) and was found to be carcinogenic in rodents given large doses by oral gavage [15]. Thus, chloroform had “sufficient evidence” for carcinogenicity to animals, being classified as group 2 according to the International Agency for Research on Cancer (IARC) [16]. This agent produced benign and malignant tumors in liver and kidney cells of mice following oral gavage (4 mmol/kg), and it was capable of inducing MN in rat kidney [17]. On the other hand, dogs were given this substance as a toothpaste as repeated exposure for long term. No association was observed between chloroform exposure and incidence of neoplasm [18].

Chloroethane is a colorless, flammable gas with an ethereal odor. It is a weak alkylating agent and has low acute toxicity, and absence of genotoxic potential has been observed below 40000 ppm [19]. No indication of mutagenicity (MN assay) was reported in male and female B6C3F1 mice exposed to high concentrations of 25000 ppm (6 h/day; 3 days). Even under very high exposure conditions, an* in vivo* genotoxic potential of chloroethane may not be a determinant factor for inducing the uterine carcinoma in the B6C3F1 mouse [20].

Trichloroethylene (TCE) started to be used as anesthetic agent in 1935, in the USA, but for a long time it is not anymore, despite the fact that it is still being used in Africa for anesthesia purpose. It is a volatile chlorinated solvent that has been commonly used as a metal degreaser and general purpose solvent in the occupational setting and has been estimated to be present in about one-third of municipal water supplies in the United States [21]. It was previously found that TCE undergoes metabolic activation in the kidney and induces DNA single-strand breaks in this organ [22, 23]. Its metabolism through a cytochrome P450 (CYP) pathway involving* CYP2E1* results in numerous metabolites, as well as some currently used halogenated anesthetics, including chloral, chloral hydrate, dichloroacetic acid, trichloroacetic acid, trichloroethanol, and trichloroethanol glucuronide, and also glutathione (GSH) conjugation metabolites [24, 25]. Several commercially anesthetic metabolites were found to be nonmutagenic, except for chloral hydrate [26]. IARC recently upgraded the carcinogenicity classification of TCE to “carcinogenic to humans” [27]. However, the carcinogenicity of this agent and its regulation is a matter of continuing debate despite the extensive database of* in vivo* animal studies. Adult male Swiss albino CD1 mice were exposed to 457 mg/kg of TCE and increased MN frequencies in polychromatic erythrocytes (PCE) were observed after 30 h of exposure in bone marrow [28]. A single dose of 4 mmol/kg of TCE increased the frequency of micronucleated rat kidney cells [17]. Contrarily, absence of genotoxicity of TCE, evaluated by comet assay, was reported in kidney male Sprague-Dawley rats exposed by inhalation 6 h per day from 500 to 2000 ppm [29]. Male C57BL/6J mice and CD rats were exposed in groups of five to target concentrations of 0, 5, 500, and 5000 ppm TCE for 6 h. Tissue samples were taken between 18 and 19 h after exposure and there were no significant increases in either sister SCE, CAs, or MN in binucleated peripheral blood cells. Cytogenetic damage was observed in the rat bone marrow, especially at the high concentration of 5000 ppm. Thus, it seems that there is a weak evidence of genotoxicity, as observed in most of the studies evaluated in rodents. High concentrations or doses of TCE can be cytotoxic and may produce toxic effects, or even carcinogenicity, especially renal tumors [30].

In relation to methoxyflurane (C_3_H_4_Cl_2_F_2_O), it was tested for carcinogenicity in mice by inhalation in utero in one limited study. No treatment-related neoplasm was observed [31]. Dichloroacetic acid, a metabolite of methoxyflurane, was found as a weak mutagen [26]. To the best of our knowledge, there is no data available for genotoxicity evaluated* in vivo* for this anesthetic.

Halothane was introduced in clinical practice in 1956, in Great Britain. It had been widely used in the past but has been mostly replaced in clinical practice by other volatile anesthetics due to its hepatotoxicity potential. Nevertheless, it is still employed in several developing countries [32]. Halothane (2% for 1 h) produced a dose-dependent increase in the rate of lethal mutations, investigated using the sex-linked recessive lethal assay in the fruit fly* Drosophila melanogaster*, but the mutagenicity was independent of the presence of the nitrous oxide [33]. Halothane (4 mmol/kg) showed positive result for MN formation in kidney cells from albino rats [17]. Two-month male Swiss albino mice were exposed to halothane at a dose of 1.5 vol% in oxygen (3 L/min) for 2 h daily for three consecutive days. The repeated exposure may enhance genetic damage since DNA damage was increased in many organs [34]. However, subsequent repair of the mentioned cells after repeated cell exposition to inhalation anesthetics remains unknown. On the other hand, halothane did not increase SCE in rodents* in vivo* or induce formation of MN or CA in bone marrow [35]. When mice were exposed in utero three times weekly for 78 weeks at the maximum tolerated dose or 24 times at several dose levels no treatment-related neoplasm was observed [31, 36]. No carcinogenic effect was reported in rats exposed to a low level of halothane [37].

Enflurane (2-chloro-1,1,2-trifluoroethyl difluoromethyl ether) did not induce genotoxic effects in renal cells of rats [17] or led to dominant lethal mutations in rodents* in vivo* [16]. The anesthetic was not found mutagenic when evaluated in the fruit fly [33]. It was tested for carcinogenicity by inhalation in one strain of mice at the maximum tolerated dose [38] and at several dose levels in a limited study in which treatment started in utero [31]. No treatment-related neoplasm was observed.

Nitrous oxide or nitrogen protoxide, also known as “laughing gas,” is a weak anesthetic agent and for this reason is usually given in combination with more powerful volatile anesthetic drugs, such as the halogenated. It still remains a source of controversy due to fears over its adverse effects. Nitrous oxide at concentrations of 40% and 80% was not mutagenic when evaluated in* Drosophila melanogaster* [33]. This gas was tested for carcinogenicity by inhalation in mice and rats. In one limited study in mice in which exposure started in utero, no treatment-related neoplasm was observed [31]. In addition, no carcinogenic effect was observed in rats chronically exposed to a low dose of nitrogen protoxide [37]. On the other hand, in concentrations greater than 50%, this agent is considered teratogenic, causing an increased incidence of fetal resorption and visceral and skeletal abnormalities, when administered to pregnant rats for 24-hour periods during organ development and when given in low concentration (0.1%) continuously to rats during pregnancy [39, 40]. Thus, scarce literature is available on genotoxicity of nitrous oxide evaluated in animal model.

Inhalation anesthetics are metabolized in animals and humans by the mixed function oxidases of liver microsomes. A plausible explanation of genotoxic potential of some inhalation anesthetics could be by the metabolism pathway, giving rise to reactive products; for example, 25% of halothane administered is metabolized. Moreover, it is suggested that halogenated anesthetics, such as halothane, may act similar to radiomimetic drugs inducing damage to the genome in any phase of the cell cycle [41]. Despite the fact that the structure of some anesthetics is chemically similar to carcinogens [26], enflurane, halothane, and nitrous oxide have inadequate evidence for carcinogenicity to animals [16]. On the other hand, the exposure to nitrous oxide increases the plasma concentrations of folate and homocysteine and decreased plasma concentrations of methionine synthase, a significant reduction of vitamin B12, which may interfere with the synthesis of nucleic acids and protein [42]. This pathway is critical to cellular function, and decreased methionine synthase activity can result in both genetic and protein aberrations [43].

## 3. Currently Used Halogenated Anesthetics

### 3.1. Isoflurane

Isoflurane (2,2,2-trifluoro-1-[trifluoromethyl]ethyl ether) started to be used in clinical practice in 1980 in USA. This anesthetic has lower blood solubility than halothane and enflurane. A disadvantage is related to the pungent odor, which limits its use in pediatric patients. Isoflurane was investigated, by inhalation, for carcinogenicity in one strain of mice. The results showed that liver tumors appeared in one study [44] but no related neoplasm was found in another study [31]. According to IARC, both experiments had limitations, showing inadequate evidence for carcinogenicity to isoflurane when evaluated in animals [16]. This anesthetic was not found to be mutagenic when evaluated at 1% or 2% when male wild-type flies (sex-linked recessive lethal assay in* Drosophila melanogaster*) were exposed for 1 h [33]. However, a few positive genotoxic effects of isoflurane have already been published. Six eight-week-old male Sprague-Dawley rats were exposed to isoflurane 1% in air for 30 or 60 min in a breathing chamber, associated or not with ethanol. DNA breaks, evaluated by the comet assay, increased in a time-dependent manner detected in lymphocytes, spleen, liver, bone marrow, and brain, except for lung cells, and alcohol induced additional DNA damage [45]. Clastogenic effect was also detected in male Sprague-Dawley albino rats (100–150 g) exposed to isoflurane in a single oral dose of 4 mmol/kg, by the increase of micronucleated kidney cells [17]. In addition, repeated exposure to isoflurane (1.7 vol% for 2 h daily; three consecutive days) in oxygen (3 L/min) induced genotoxicity in leukocytes and cells from brain, liver, and kidney of male Swiss albino mice (8 weeks old, 20–25 g) [34]. The same authors reported that, in comparison with halothane (1.5% at the same conditions), isoflurane produced a significant lower genotoxic effect, especially in liver and brain tissues. It is believed that whether isoflurane or halothane can react with DNA, the most probable alkaline-labile modification may be an alkylation at the N7 position of purines. However, there is no data so far showing this hypothesis [46]. Another hypothesis is that residual metabolic oxidation/reduction of anesthetics increases reactive products [34, 46]. However, it must be highlighted that hepatic biotransformation of isoflurane is low (≤0.2%).

### 3.2. Sevoflurane

The third generation of inhaled halogenated anesthetics consists of sevoflurane and desflurane, which have several properties that make them potentially useful as anesthetic. Sevoflurane (fluoromethyl 2,2,2-trifluoro-1-[trifluoromethyl]ethyl ether) has a sweet smell, being widely used in children.

Sevoflurane has not undergone formal testing but was approved for clinical use by the US Food and Drug Administration (FDA) presumably because of the lack of carcinogenicity associated with the group of inhaled agents currently in use [47]. Only a few studies have evaluated DNA damage in animals repeatedly exposed to sevoflurane. New Zealand male rabbits were anesthetized with 3% concentration of sevoflurane (1.4 MAC) with 4 L/min in oxygen for 3 h/day for three days [48]. The authors found similar DNA damage levels in lymphocytes before anesthesia (baseline), on the first and fifth day of anesthesia. However, increased genetic damage was observed on the second and third day after repeated anesthesia. Swiss albino mice exposed to 2 h daily for three days to sevoflurane (2.4 vol% of a 50 : 50 mixture of oxygen and air at 3 L/min) showed increase of DNA damage in brain and liver cells after 6 h of the last exposure whereas leukocytes and kidney cells presented the significant increase after 24 h. In addition, it was reported that MN frequency in peripheral blood reticulocytes was the highest 6 h after the last sevoflurane exposure [49]. Moreover, increase of micronucleated kidney cells was detected after a single oral dose of 4 mmol/kg of sevoflurane in male Sprague-Dawley albino rats [17]. It was pointed out that this dose is approximately 1/7 of the oral LD_50_ in rats for enflurane; the corresponding LD_50_ for sevoflurane is not available, but data on its inhalation toxicity suggest that it should be similar to enflurane. Sevoflurane undergoes a moderate degree of metabolism (about 5%) and can form a toxic product known as compound A, a vinyl ether. However, this compound did not increase the number of MN in bone marrow cells from male mice (8 weeks old) exposed for 3 h at concentration of 150 ppm [50]. Thus, considering the few data on this issue, sevoflurane seems to contribute to inducing DNA lesions* in vivo*. The genetic damage seems to be repaired within the consecutive days of the last exposure.

Sevoflurane is metabolized in the liver by cytochrome P450 and the metabolites could lead to DNA damage. However, it must be emphasized that there are only a few reports in the literature about genotoxicity of sevoflurane and the studies are very different; that is, they have used different animal models and species, with different times of exposure and routes, different doses, and different time points evaluated besides a variety of endpoints evaluated, making the comparison among the papers difficult. Thus, further studies must be done to clarify whether sevoflurane and their metabolites can be genotoxic. Moreover, other possible confounding factors such as temperature, hemodynamic data, and air flow rate can interfere in the results.

### 3.3. Desflurane

Desflurane (1,2,2,2-tetrafluoroethyl difluoromethyl ether) differs from isoflurane in the substitution of the chlorine atom by fluorine. It has a low blood/gas solubility coefficient that allows rapid changes in anesthesia level. High vapor pressure, ultrafast duration, and moderate potency are the main characteristics of this agent [51]. It has the highest minimum alveolar concentration, MAC (6%-7%), among the halogenated anesthetics [52]. This anesthetic has minimal hepatic biotransformation (0.02%) and has not undergone formal testing but was approved for clinical use by FDA [53] presumably because of the lack of carcinogenicity associated with the group of volatile anesthetics currently in use. The only report, to the best of our knowledge, is that desflurane showed negative result for* in vivo* cytogenetics [54]. Thus, being one of the newer anesthetics, there is still missing data about the possible genotoxic potential of desflurane evaluated in animals. It is worthy to focus research on evaluation of genotoxicity and mutagenicity of desflurane* in vitro*, in animals and also in humans.

Several studies have been designed to demonstrate toxicity of volatile anesthetics in animals. None of the used inhaled volatile anesthetic agents, such as isoflurane, sevoflurane, and desflurane, has been shown to cause severe adverse effects with clinical or trace exposure levels, in either the short or long term [47].

Many factors must be considered before assessing the risk of inhaled anesthetics to humans. Species variations in drug metabolism and toxicity, different dosages and exposure times, the flow rate (in air or in oxygen), and hypoxia during anesthetic procedure are some of the factors that make it difficult to extrapolate results from one species to another from* in vivo* situations to humans [33]. However, the knowledge that one anesthetic can damage DNA in a number of genotoxic and mutagenic testes should be considered when assessing the overall toxicity of this anesthetic in human population. Volatile anesthetics are classified as group 3 in terms of their carcinogenicity by IARC [16].

## 4. Intravenous Anesthetics

It is known that propofol has a phenolic group in its chemical structure, similar to some antioxidant compounds, and it seems not to be genotoxic and even prevents possible DNA damage in patients [55–57]. Only a few studies have evaluated possible genotoxic effect of propofol (2,6 diisopropylphenol) in experimental studies. This anesthetic presented negative results for* in vivo* cytogenetics [54].

For environmental purposes, propofol has been successfully applied intravenously [58–60] and by immersion bath [61, 62] in fish. The lack of genotoxicity (comet assay) and mutagenicity (MN assay) was demonstrated in Nile tilapia exposed to this anesthetic by immersion bath [63].

Literature data on genotoxicity activity of benzodiazepines are scarce, restricted to few of them, and contradictory. Alprazolam was found not to be mutagenic in* in vivo* cytogenetics study, but diazepam showed positive result [54]. There are no reports about genotoxicity of etomidate or the barbiturate thiopental evaluated in animal models. Pentobarbital showed positive results* in vivo*, whereas phenobarbital sodium did not lead to SCE or CA in animal model but was shown to produce tumors in mice and also enhanced DNA damage (comet assay) in liver from rodents [16, 64, 65].

In relation to ketamine, antioxidative properties have already been described [66]. Ketamine can prevent brain damage (DNA fragmentation) in rats after ischemia and reperfusion [67]. On the other hand the administration of ketamine in a clinically relevant single dose induced apoptosis in neonatal mouse brain [68]. Induction of anesthesia using a combination of drugs for veterinary anesthesia purpose (including ketamine, diazepam, xylazine, and isoflurane at 1.3% in 100% oxygen) did not lead to genetic damage, detected by comet assay, when comparing before and 1 and 24 h after the induction of anesthesia in pony and horses [69].

Opioids codeine, dextromethorphan, and dextropropoxyphene, given orally to Swiss albino mice, were devoid of genotoxicity by MN and comet assays [12]. Remifentanil showed negative result when evaluated by cytogenetic test [54]. Literature shows that morphine was nonmutagenic in the* Drosophila melanogaster* sex-linked recessive lethal mutation assay. However, it increased MN frequency in both red blood and bone marrow cells in the mouse [70]. Some hypothesis of the* in vivo* clastogenic effects reported with morphine in mice may be directly related to increase in glucocorticoid levels produced by morphine in this species [71] or a consequence of hypothermia [72], which is caused in rodents by this drug, but this is worthy of further study. Literature data on* in vivo* genotoxic potential of benzodiazepines and other drugs used during anesthesia are scarce.

Most experimental tests are performed using only one anesthetic agent. However, the use of a single anesthetic is a rare occurrence in clinical practice. A combination of inhaled anesthetic gases and intravenous drugs is usually delivered during general anesthesia; this practice is called balanced anesthesia and is used as it takes advantage of the beneficial effects of each anesthetic agent to reach surgical anesthesia. But this may theoretically potentate their genotoxic effects. Given that different anesthetic drugs are used in combination, it is hard to understand if effects or absence of effects is related to an individual agent action or a synergy action of different anesthetics involved.

## 5. Local Anesthetics

The most used local anesthetics are lidocaine, articaine, and prilocaine. Despite the utility of these agents in many procedures, including the dental medicine, few data are available on their possible genetic toxicity. Lidocaine showed negative results when evaluated in* Drosophila melanogaster*, using the wing somatic mutation and recombination test (SMART), related to gene and chromosomal mutation, or reciprocal recombination, and* in vivo* cytogenetic test [54, 73]. Articaine was also unable to induce mutagenicity in* Drosophila*, whereas prilocaine, but not its metabolites, displayed genotoxic activity by causing homologous recombination [73].

Centbucridine (4-N-butylamino-1,2,3,4-tetrahydroacridine hydrochloride) is a local anesthetic that did not show genotoxic effect when DNA breaks were evaluated in liver cells or by CA and SCE in bone marrow cells following a single acute exposure in mouse model [74].

Benzocaine (ethyl r-aminobenzoate) was first synthesized in 1890 as a local anesthetic. Its genotoxicity was investigated at 500, 1000, and 2000 mg/kg in male mice. Bone marrow MN did not indicate mutagenic effect of this drug [75]. In addition to its use in human medicine, benzocaine is widely used in aquaculture, albeit it does not have FDA approval for this purpose in the USA. Anesthesia of fish during aquatic biomonitoring or laboratory studies contributes to improving fish welfare. In this species, blood erythrocytes are mainly used as sentinel markers of genotoxic exposure. This anesthetic (80 mg/L) did not show a genotoxic effect, using the comet assay, when evaluated in Nile tilapia [76].

Tricaine methanesulfonate (MS-222) is one of the most important and commonly used anesthetics on fish and is approved by the FDA for use on aquatic organisms [77]. This agent is considered to be a local anesthetic, although it acts systemically in fish. Bath exposure with MS-222 was not considered genotoxic, when detected by comet assay, when evaluated in juvenile fish [78]. Besides, MS-222 and benzocaine are analogues of procaine (typically used as local anesthetics in humans). Another procaine analogue, carbisocaine, has been shown to have genotoxic activity [79].

## 6. Summary

In this review, we have highlighted the genotoxic potential of some anesthetics and drugs used in anesthesia. Although their genotoxicity and possible action mechanisms have been proposed, much remains to be examined, since conflicting and a few results are available in the literature. It must be emphasized that the protocols used are quite different, making the comparisons and conclusions difficult. In addition, the role of these agents concerning the interference on cellular signal pathways, gene expression profiles, and epigenetic mechanisms is fundamental for elucidating putative interactions with cellular machinery. Therefore, this is an area that warrants investigation.
